# Three-Dimensional Segmented Poincaré Plot Analyses SPPA3 Investigates Cardiovascular and Cardiorespiratory Couplings in Hypertensive Pregnancy Disorders

**DOI:** 10.3389/fbioe.2014.00051

**Published:** 2014-11-12

**Authors:** Claudia Fischer, Andreas Voss

**Affiliations:** ^1^Department of Medical Engineering and Biotechnology, University of Applied Sciences Jena, Jena, Germany

**Keywords:** multivariate analysis, hypertensive pregnancy disorders, heart rate variability, Poincaré plot analysis, risk stratification, non-linear dynamics, blood pressure variability, respiration

## Abstract

Hypertensive pregnancy disorders affect 6–8% of gestations representing the most common complication of pregnancy for both mother and fetus. The aim of this study was to introduce a new three-dimensional coupling analysis methods – the three-dimensional segmented Poincaré plot analyses (SPPA3) – to establish an effective approach for the detection of hypertensive pregnancy disorders and especially pre-eclampsia (PE). A cubic box model representing the three-dimensional phase space is subdivided into 12 × 12 × 12 equal predefined cubelets according to the range of the SD of each investigated signal. Additionally, we investigated the influence of rotating the cloud of points and the size of the cubelets (adapted or predefined). All single probabilities of occurring points in a specific cubelet related to the total number of points are calculated. In this study, 10 healthy non-pregnant women, 66 healthy pregnant women, and 56 hypertensive pregnant women (chronic hypertension, pregnancy-induced hypertension, and PE) were investigated. From all subjects, 30 min of beat-to-beat intervals (BBI), respiration (RESP), non-invasive systolic (SBP), and diastolic blood pressure (DBP) were continuously recorded and analyzed. Non-rotated adapted SPPA3 discriminated best between hypertensive pregnancy disorders and PE concerning coupling analysis of two or three different systems (BBI, DBP, RESP and BBI, SBP, DBP) reaching an accuracy of up to 82.9%. This could be increased to an accuracy of up to 91.2% applying multivariate analysis differentiating between all pregnant women and PE. In conclusion, SPPA3 could be a useful method for enhanced risk stratification in pregnant women.

## 1 Introduction

Hypertensive pregnancy disorders are leading causes of maternal and fetal morbidity and mortality and affect 6–8% of all pregnancies (NHBPEP, 2000). The classification of the hypertensive pregnancy disorders was performed according to the guidelines of the “National High Blood Pressure Education Program Working Group on High Blood Pressure in Pregnancy” (NHBPEP, 2000). Thereafter, hypertension in pregnancy is related to one of four conditions: (1) chronic hypertension (CH) predating pregnancy; (2) pre-eclampsia (PE) as a serious, systemic syndrome of elevated blood pressure, proteinuria, and other influences; (3) CH with superimposed PE; and (4) pregnancy-induced hypertension (PIH) (NHBPEP, 2000; Zamorski and Green, 2001). Several studies suggest that the autonomic nervous system plays an important role in the process of developing hypertensive pregnancy disorders, especially PE. A sympathetic overactivity in women with GH (Greenwood et al., 1998) and PE (Schobel et al., 1996) compared to healthy pregnant and hypertensive non-pregnant women has been proven using the technique of microneurography. Furthermore, it is well known that maternal autonomic cardiovascular control is strongly affected by pregnancy (Hermida et al., 1997).

In recent years, several studies have demonstrated that non-linear methods provide additional diagnostic and prognostic information representing a useful complement to traditional time- and frequency-domain analyses (Voss et al., 2009; Riedl et al., 2010). Therefore, Voss et al. (2000) reported significant differences in heart rate variability (HRV) and spontaneous baroreflex sensitivity (BRS) between non-pregnant and normal pregnant women applying standard linear as well as non-linear parameters. Furthermore, it was shown that the differences in HRV and BRS depend on the stage of gestation. Faber et al. (2004) demonstrated that HRV and blood pressure variability (BPV) reveal significant alterations during the development of hypertensive disorders. The Poincaré plot analysis (PPA) provides a visual tool to characterize the complex nature of time series fluctuations (Kamen and Tonkin, 1995). Parameters from PPA were shown to be powerful predictors of postoperative ischemia (Laitio et al., 2002). However, the traditional PPA parameters lose most of the non-linear information contained in a time series (Brennan et al., 2002; Guzik et al., 2007; Karmakar et al., 2009).

Therefore, Voss et al. (2010a) introduced the segmented Poincaré plot analysis (SPPA) method, which constitutes an enhancement of the traditional PPA. The SPPA method captures the non-linear characteristics of a time series, and therefore, overcomes several limitations of traditional PPA method, i.e., the high correlation between the PPA indices and linear parameters (Guzik et al., 2007). For example, SPPA indices derived from beat-to-beat interval time series were able to discriminate between low and high risk patients with dilated cardiomyopathy, which was not possible when applying traditional PPA.

Voss et al. (2010b) already proved the prediction of hypertensive pregnancy disorders applying the bivariate joint symbolic dynamics (JSD) method introduced by Baumert et al. (2002). As one result, they showed that the cardiovascular regulatory system was changed considerably depending on the type of hypertensive disorder. Analyzing the couplings between heart rate and blood pressure time series with JSD led to a significant differentiation between chronic or PIH and PE. However, the influences of normal pregnancy and pregnancy disorders on the cardiorespiratory system were not considered.

The objectives of this study were to introduce varying enhancements of the new multivariate coupling method (varying positions of the cloud of points as well as different dimensions of the cubelets within the cubic box model) and to most suitable indices differentiating between hypertensive pregnancy disorders and PE. Therefore, the new three-dimensional segmented Poincaré plot analysis (SPPA3) is introduced representing PPA using a cubic box model. This is based on first ideas published in Khandoker et al. (2013). We investigated couplings between the subsystems of cardiovascular and cardiorespiratory autonomic regulation applying time series of beat-to-beat intervals (BBI), systolic blood pressure (SBP), diastolic blood pressure (DBP), and respiration rate (RESP). Furthermore, we compared an univariate approach of SPPA3 [using time shifts of only one subsystem (BBI, SBP, DBP or RESP)] with the multivariate SPPA3 to check if the multivariate approach further improves risk stratification in pregnant women.

## 2 Materials and Methods

### 2.1 Patients

In this study, we enrolled data from 112 pregnant women (mean age: 28 years, range: 19–38 years, SD: 5.1 years) from the University Hospital Leipzig. Sixty-six of them had normal pregnancies (PREG), 13 suffered from CH, 14 from pregnancy-induced hypertension (PIH), and 19 developed a pre-eclampsia (PE). For more details, see Table 1.

**Table 1:** **Data of pregnant women and controls**.

Group	Number	Age – mean ± SD (years)	Week of gestation		
			Mean	Range	SD
CON	10	26.9 ± 2.6	–	–	–
PREG	66	28.1 ± 4.9	34	19–40	4.7
CH	13	28.6 ± 5.2	30	20–39	6.7
PIH	14	27.5 ± 5.1	35	27–39	3.5
PE	19	27.6 ± 6.0	32	25–39	3.9
CH + PIH	27	28.0 ± 5.1	33	20–39	5.6
CH + PIH + PE	46	27.9 ± 5.4	32	20–39	4.9
PREG + CH + PIH	93	28.1 ± 4.9	33	19–40	5.0

*Non-pregnant women as controls – CON, normal pregnancies – PREG, chronic hypertension – CH, pregnancy-induced hypertension – PIH, pre-eclampsia – PE*.

As a control group, 10 age-matched healthy women (CON; mean age 26.9 years, range 24–32 years, SD 2.6 years) from the Department of Medical Engineering and Biotechnology, University of Applied Sciences Jena were investigated. None of these controls had a cardiovascular or renal disease or took medications with cardiovascular effects.

The investigation conforms to the principles outlined in the Declaration of Helsinki. Local ethics committee approval and informed consent of all subjects were provided.

### 2.2 Signal acquisition and preparation

Thirty minutes of continuous blood pressure (NIBP, fs = 200 Hz, resolution = 0.1 mmHg) and breathing intervals (RESP – respiration belt) were recorded in supine position during the late morning hours. NIBP was measured on the left middle finger applying the non-invasive Portapres M2 blood pressure monitor [TNO-TPD, Amsterdam, Netherlands (Wesseling, 1996)].

The time series of BBI, SBP, and DBP were extracted using the “BeatFast” pattern recognition software package (TNO Biomedical Instrumentation, The Netherlands). The maxima of the respiratory curve were detected to get the respiratory frequency (breathing cycle length). Ectopic beats and other disturbances were excluded and interpolated by an adaptive variance estimation algorithm, considering the variance within the time series just before and directly after the event (Wessel et al., 2000).

### 2.3 Three-dimensional segmented Poincaré plot analysis

The new three-dimensional SPPA3 method is a multivariate analysis technique based on the SPPA method introduced by Voss et al. (2010a). SPPA method retains non-linear features from a system based on the traditional PPA (Kamen and Tonkin, 1995; Brennan et al., 2002) by plotting BBI time series over systolic or diastolic NIBP time series. The cloud of points is segmented into 12 × 12 equal rectangles whose size (height and width) depends on the SD of BBI and NIBP time series. The number of points within each rectangle related to the total number of points was counted to get the single point probabilities. Based on these single point probabilities, two segmentation algorithms are used: summarizing all single probabilities of rows and columns.

However, SPPA3 method investigates the coupling of three time series determining both couplings between two (BBI, diastolic and systolic NIBP or RESP, diastolic and systolic NIBP) and three (BBI, diastolic NIBP, RESP or BBI, systolic NIBP, RESP) different systems represented by the time series of BBI, SBP/DBP, and RESP and plotted against each other applying a cubic box model.

#### 2.3.1 Varying positions of the cloud of points

SPPA3 considers two varying positions of the cloud of points:

- Non-rotated version: non-rotated position of the cloud of points
- Rotated version: normalization of the original cubic box model by rotating the cloud of points in each plane. This is provided by fitting linear regression lines in each plane of the cubic box model calculating the angles α between the *x*-axis and the related regression line, β between the *y*-axis and the related regression line, and γ between the *z*-axis and the related regression line. The cloud of points is rotated by the angle α (β, γ) around the main focus of the cloud of points in each plane.

#### 2.3.2 Predefined and adapted SPPA3

**Figure 1:**
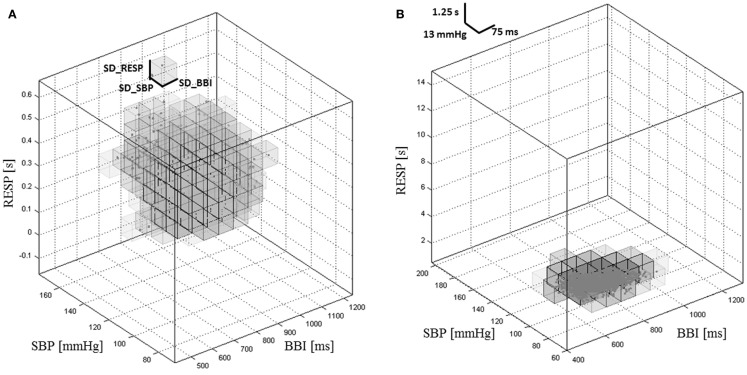
**Cubic box model of the adapted SPPA3 (A) and predefined SPPA3 (B) investigating the coupling between BBI, SBP, and RESP in a healthy pregnant woman**.

Considering non-rotated and rotated version of SPPA3, the cubic box model is subdivided into 12 × 12 × 12 equal dimensioned cubelets for a total number of *N* = 1728 cubelets – the highly segmented cubic box model. SPPA3 considers two approaches differing in the dimension of the cubelets calculated for both the rotated and non-rotated position of the cloud of points:

(1) Adapted SPPA3 (e.g., Figure 1A): this approach includes the calculation of the SD (e.g., SD_BBI) of each investigated time series. The size of each cubelet is adapted to the calculated SD with regard to the axis. The resulting 3D cubic box model consists of 12 × 12 × 12 equal cubelets whereby the center of the 3D cubic box model represents the main focus of the cloud of points.
(2) Predefined SPPA3 (e.g., Figure 1B): the 3D cubic box model represents the basic model with regard to all patients. The detailed information of the chosen borders for a single cubelet is shown in Table 2.

**Table 2:** **Definition of the cubelets’ dimensions**.

Signal	Size of a single cubelet	Range of axis	
		Min	Max
*BBI*	75 ms	400	1300
*SBP*	13 mmHg	50	206
*SDBP*	9 mmHg	22	130
*RESP*	1.25 s	0.5	15

For each cubelet, the probability of occurrence (Prob) of data points within the cubelet is calculated as:

$$\begin{array}{lll} \text{Prob }\left(X_{\text{r}},\;Y_{\text{c}},\;Z_{\text{d}}\right)=\sum \left(\text{data points}\right)∕N & \; & \\ \text{with }r,\;c,\;d=1\;\ldots \;12 & \; & \end{array}$$

*X* represents the values within each row (*r*) mapping the first coupled time series (e.g., BBI), *Y* that of each column (*c*) mapping the second coupled time series (e.g., SBP/DBP), and *Z* the values of depth (*d*) mapping the third coupled time series (e.g., RESP). Therefore, the indices *r* (row), *c* (column), and *d* (depth) name the coordinates of the specific cubelet ranging between 1 and 12. The index of each cubelet is generally defined as:

$$\begin{array}{c} X_{\text{r}}\text{\_}Y_{\text{c}}\text{\_}Z_{\text{d}}\\ \left(\text{e}.\text{g}.,\text{ BBI1\_SBP4\_RESP2 that defines the cubelet with the coordinates }r=\text{1},c=\text{4},d=\text{2}\right) \end{array}$$

#### 2.3.3 Selection of enlarged cubelet sizes

**Figure 2:**
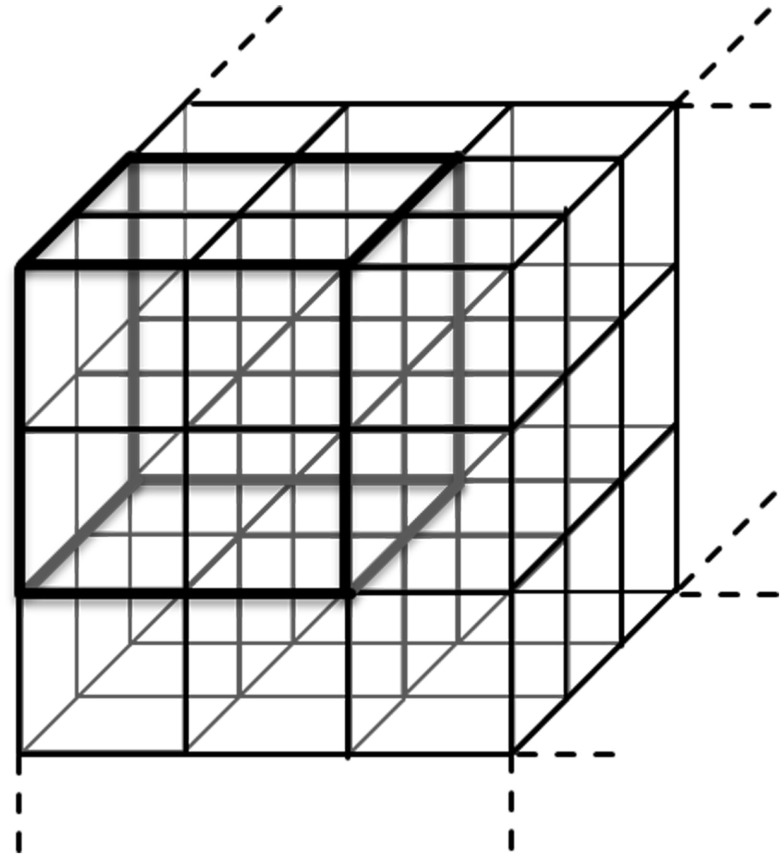
**Principle of the coarsely segmented cubic box model combining each eight cubelets to get the 6 × 6 × 6 cubic box model**.

Additionally, a further segmentation algorithm (Figure 2) was performed to calculate SPPA3 indices in a 6 × 6 × 6 cubic box model (combining each eight neighboring cubelets from the 12 × 12 × 12 cubic box model) – coarsely segmented cubic box model.

### 2.4 Univariate 3D segmented Poincaré plot analysis (univariate SPPA3)

To retain non-linear features from a system applying PPA, an enhanced pseudo phase space quantification method was introduced by Voss et al. (2010a) – the SPPA. The new univariate SPPA3 is based on SPPA enhanced by one additional embedding dimension plotting beat-to-beat time series as a function of the consecutive ones. Like multivariate SPPA3, univariate SPPA3 can be adapted to varying positions of the cloud of points (see Section 2.3.1) as well as the predefined and adapted SPPA3 (see Section 2.3.2).

Univariate SPPA3 works as following:

(1) The SD [SD(*x*)] are calculated by the traditional PPA as usually, whereas Var is the variance, *x*_n_ is a beat-to-beat interval time series (BBI, SBP, DBP or RESP) with *n* = 1, …, *N* − 2 (*N* is the length of time series), *x*_n + 1_ is the same time series shifted by a lag of τ = 1, and *x*_n + 2_ shifted by a lag of τ = 2 (Piskorski and Guzik, 2005):
  $$\begin{array}{llll} x_{\text{n}} & =\left(x_{\text{1}},\;x_{\text{2}},\ldots ,\;x_{\text{N}-\text{2}}\right)\; & & \;\\ x_{\text{n}+\text{1}} & =\left(x_{\text{2}},\;x_{\text{3}},\ldots ,\;x_{\text{N}-\text{1}}\right)\; & & \;\\ x_{\text{n}+\text{2}} & =\left(x_{\text{3}},\;x_{\text{4}},\ldots ,\;x_{\text{N}}\right)\; & & \;\\ \text{SD}\left(x_{\text{n}}\right) & =\sqrt{\text{Var }\left(x_{\text{n}}\right)}\; & & \; \end{array}$$
(2) Applying the rotated version, cloud of points is rotated at the angle α = 45° around the main focus of the plot in each plain, otherwise the original position of the cloud of points is retained.
(3) A grid of 12 × 12 × 12 cubelets is drawn into the plot starting from the main focus of the cubic box model whereas the size of each cubelet (height, width, depth) is adapted to SD(*x*_n_), SD(*x*_n + 1_), and SD(*x*_n + 2_).
(4) For each cubelet, the percentage of occurrence (Prob) of data points is calculated as:
  $$\text{Prob }\left(X_{\text{r}},\;X_{\text{c}},\;X_{\text{d}}\right)=\sum \left(\text{data points}\right)∕N$$
  *X* represents the axis of the cubic box model with *r* (row − *x*_n_), *c* (column − *x*_n + 1_), and *d* (depth − *x*_n + 2_). Therefore, the index of each cubelet is generally defined as following:
  $$\begin{array}{c} X_{\text{r}}\text{\_}X_{\text{c}}\text{\_}X_{\text{d}}\\ \left(\text{e}.\text{g}.,\text{ BBI1\_BBI4\_BBI2 that defines the cubelet with the coordinates }r=\text{1},c=\text{4},d=\text{2}\right) \end{array}$$

### 2.5 Statistical tests

The non-parametric Mann–Whitney *U*-test (SPSS Statistics 21) was performed to figure out significant differences between all investigated groups of patients for all kinds of couplings between the systems. Three levels of significance were considered prior to the presentation of the results: significant (0.01 ≤ *p* < 0.05), highly significant (*p* < 0.01), and significances fulfilling the Bonferroni criterion [*p* < 0.00003 (BF)] because of the high number of considered variables (1728).

The following group tests were performed:

- Test I: CON vs. PREG
- Test II: PREG vs. CH
- Test III: PREG vs. PIH
- Test IV: PREG vs. PE
- Test V: CH vs. PIH
- Test VI: CH vs. PE
- Test VII: PIH vs. PE
- Test VIII: CH and PIH vs. PE
- Test IX: PREG, CH and PIH vs. PE
- Test *X*: PREG vs. CH and PIH and PE.

The receiver operating characteristic (ROC) curves together with estimations for the area under the ROC curve (AUC) were computed for each single index (univariate) as well as for index sets consisting of two indices (multivariate). Therefore, discriminant analyses with both one and two indices were performed applying leave-one-out cross-validation. This served as a starting basis for the succeeding ROC analysis. The sensitivity (SENS) and specificity (SPEC) were estimated from the nearest point to 1 on the horizontal axis of each ROC.

## 3 Results

For a first evaluation of the SPPA3 (12 × 12 × 12 cubelets) method, the number of highly significant indices (*p* < 0.01) for each coupling and all types of group tests are shown in Table 3, presenting the results from the rotated versions, and Table 4 presents the results from the non-rotated versions of SPPA3. In general, it could be stated that non-rotated predefined SPPA3 leads to most significant cubelets especially for couplings between the three different systems (BBI, SBP, and RESP) except test II and V. The highest number of significant cubelets was revealed from test IV (PREG vs. PE).

**Table 3:** **SPPA3 with rotation of cloud of points**.

Test	I	II	III	IV	V	VI	VII	VIII	IX	X
**Adapted SPPA3**
BBI_DBP_RESP	18	13	13	36	3	1	5	3	32	7
BBI_SBP_DBP	11	54	23	13	2	4	19	15	10	28
BBI_SBP_RESP	20	18	26	49	3	0	18	9	37	24
SBP_DBP_RESP	14	2	7	13	0	0	4	8	14	2
**Predefined SPPA3**
BBI_DBP_RESP	33	0	2	24	2	4	6	11	24	9
BBI_SBP_DBP	108	1	27	45	2	17	20	16	42	15
BBI_SBP_RESP	25	4	7	42	0	1	6	9	36	24
SBP_DBP_RESP	30	19	9	56	0	5	1	1	47	20

*Number of significant cubelets (*p* < 0.01) for the two approaches of SPPA3 (adapted and predefined) applying all kinds of couplings; the couplings yielding the most significant cubelets are marked*.

**Table 4:** **SPPA3 without rotation of the cloud of points**.

Test	I	II	III	IV	V	VI	VII	VIII	IX	X
**Adapted SPPA3**
BBI_DBP_RESP	22	3	13	13	2	4	11	8	17	2
BBI_SBP_DBP	34	3	5	24	1	10	20	25	35	1
BBI_SBP_RESP	44	2	9	39	1	10	29	34	44	1
SBP_DBP_RESP	21	4	7	19	1	8	18	22	25	1
**Predefined SPPA3**
BBI_DBP_RESP	112	0	20	110	3	21	15	39	109	46
BBI_SBP_DBP	105	17	21	94	6	23	26	38	94	59
BBI_SBP_RESP	128	4	29	143	1	25	45	55	130	112
SBP_DBP_RESP	61	18	11	83	9	24	14	26	73	63

*Number of significant cubelets (*p* < 0.01) for the two approaches of SPPA3 (adapted and predefined) applying all kinds of couplings; the couplings yielding the most significant cubelets are marked*.

In clinical routine, the most important tasks are to differentiate between PE and the other hypertensive disorders (including CH and PIH) and between PE and all other pregnancies (including PREG, CH, and PIH). Therefore, only the results of tests VIII and IX were further presented.

The best results from multivariate SPPA3 analysis (tests VIII and IX) applying rotated and non-rotated approaches are shown in Table 5 (12 × 12 × 12 cubelets for the cubic box model).

**Table 5:** **Best result of each approach including rotated and non-rotated methods for the highly segmented cubic box model (12 × 12 × 12 cubelets)**.

Meth	ROT	Test	Index	*U*-test	SENS	SPEC	AUC	VIII: CH, PIH; IX: PREG, CH, PIH		PE	
								Mean	SD	Mean	SD
Predefined SPPA3	Yes	VIII	BBI3_DBP8_RESP5	**	47.4	96.3	71.7	0.096	0.498	2.492	5.238
			BBI7_SBP10_DBP9	**	15.8	100.0	60.5	0.000	0.000	0.473	1.550
			BBI3_SBP9_RESP4	**	47.4	92.6	70.2	0.192	0.691	3.658	8.019
			SBP2_DBP2_RESP3	**	36.8	96.3	65.1	0.160	0.823	1.532	3.358
		IX	BBI3_DBP8_RESP5	***	47.4	96.8	72.2	0.069	0.464	2.492	5.238
			BBI3_SBP7_DBP3	***	21.1	100.0	60.5	0.000	0.000	0.473	1.550
			BBI3_SBP9_RESP4	***	47.4	97.8	72.7	0.056	0.378	3.658	8.019
			SBP4_DBP3_RESP11	***	31.6	100.0	65.8	0.046	0.444	1.532	3.358
	No	VIII	BBI4_DBP8_RESP7	**	57.9	92.6	74.3	0.481	2.421	2.645	5.780
			BBI8_SBP10_DBP8	**	100.0	63.0	81.5	2.582	4.379	0.000	0.000
			BBI8_SBP11_RESP4	**	100.0	63.0	80.5	3.619	7.233	0.003	0.013
			SBP7_DBP8_RESP7	**	89.5	74.1	82.6	5.718	11.486	0.386	1.657
		IX	BBI2_DBP9_RESP5	***	42.1	98.9	70.4	0.000	0.000	0.103	0.283
			BBI1_SBP8_DBP2	***	63.2	94.6	79.3	0.032	0.312	0.814	1.886
			BBI3_SBP8_RESP7	***	47.4	100.0	73.7	0.124	0.987	2.326	5.746
			SBP2_DBP2_RESP12	***	78.9	80.6	78.1	0.000	0.000	0.289	0.769
Adapted SPPA3	Yes	VIII	BBI7_DBP5_RESP5	**	94.7	18.5	52.2	0.031	0.102	0.009	0.021
			BBI9_SBP6_DBP12	***	78.9	88.9	82.6	0.017	0.049	0.171	0.243
			BBI6_SBP6_RESP12	**	89.5	51.9	71.3	2.872	1.269	3.951	1.489
			SBP5_DBP4_RESP5	**	57.9	63.0	57.9	0.089	0.170	0.107	0.182
		IX	BBI6_DBP9_RESP2	**	30.4	100.0	65.2	0.000	0.000	0.009	0.021
			BBI9_SBP6_DBP12	***	31.6	96.8	64.2	0.051	0.175	0.171	0.243
			BBI6_SBP9_RESP1	**	73.7	68.8	76.4	0.002	0.014	0.032	0.060
			SBP6_DBP7_RESP10	**	52.6	88.2	70.3	0.903	0.760	0.367	0.286
	No	VIII	BBI3_DBP5_RESP2	**	78.9	77.8	80.7	0.106	0.103	0.018	0.042
			BBI9_SBP5_DBP11	**	68.4	92.6	80.7	0.006	0.018	0.086	0.085
			BBI5_SBP7_RESP12	**	84.2	81.5	82.8	1.050	0.578	1.794	0.566
			SBP8_DBP9_RESP9	**	68.4	85.2	81.1	0.082	0.144	0.306	0.257
		IX	BBI7_DBP3_RESP3	**	68.4	84.9	79.9	0.009	0.025	0.071	0.132
			BBI9_SBP6_DBP11	***	78.9	80.6	82.9	0.072	0.210	0.313	0.324
			BBI5_SBP7_RESP12	***	78.9	74.2	80.1	1.106	0.550	1.794	0.566
			SBP8_DBP9_RESP9	***	47.4	96.8	72.2	0.090	0.155	0.306	0.257

Mann–Whitney *U*-test and discriminant analysis (SENS – sensitivity, SPEC – specificity, AUC – area under the ROC curve) of the most important group tests (CH and PIH vs. PE – Test VIII and PREG, CH and PIH vs. PE – Test IX) including mean value and SD of investigated groups; highest AUC values (AUC > 80%) are marked [**p < 0.01, ***p < 0.00003 (BF); ROT – rotation].

The most discrimination power was shown for non-rotated adapted SPPA3 applying couplings between two (Test IX – BBI9_SBP6_DBP11; AUC = 82.9%, SENS = 78.9%, SPEC = 80.6%) and three coupling systems (Test VIII – BBI5_SBP7_RESP12; 82.8%/84.2%/81.5%) and the non-rotated predefined SPPA3 applying couplings between two coupling systems (Test VIII – SBP7_DBP8_RESP7; 82.6%/89.5%/74.1%).

The best results of univariate SPPA3 analysis for the rotated as well as non-rotated approaches are shown in Table 6. For the group tests, PREG, CH, and PIH vs. PE (test IX) the Bonferroni criterion was fulfilled. The best result [*p* < 0.00003 (BF)] applying non-rotated predefined SPPA3 analysis was achieved by BBI8_BBI3_BBI2 (63.4%/31.6%/95.1%) and applying the rotated version of predefined SPPA3 analysis by BBI9_BBI3_BBI2 (60.5%/21.1%/100%) for test IX.

**Table 6:** **Most significant results of Mann–Whitney *U*-test as well as its discriminant analysis of univariate SPPA3 applying all kinds of approaches for the most important group tests VIII (CH, PIH vs. PE) and IX (PREG, CH, PIH vs. PE)**.

Index	Test		DA			PREG		CH		PIH		PE	
	VIII	IX	SENS	SPEC	AUC	Mean	SD	Mean	SD	Mean	SD	Mean	SD
**Non-rotated predefined SPPA3**													
BBI8_BBI3_BBI2	–	***	31.6	95.1	63.4	0.011	0.076	0.000	0.000	0.000	0.000	0.315	0.952
BBI7_BBI1_BBI10	–	***	21.1	98.1	59.5	0.000	0.000	0.000	0.000	0.000	0.000	0.167	0.665
**Rotated predefined SPPA3**													
BBI9_BBI3_BBI2	–	***	21.1	100.0	60.5	0.000	0.000	0.000	0.000	0.000	0.000	0.313	1.196
**Non-rotated adapted SPPA3**													
SBP5_SBP5_SBP2	***	–	78.9	80.6	86.6	1.789	0.538	1.798	0.598	1.879	0.465	2.745	0.762
SBP5_SBP5_SBP2	–	***	78.9	80.6	86.6	1.789	0.538	1.798	0.598	1.879	0.465	2.745	0.762
**Rotated adapted SPPA3**													
SBP5_SBP5_SBP11	***	–	84.2	68.8	80.9	0.088	0.077	0.111	0.089	0.131	0.083	0.021	0.029
SBP2_SBP3_SBP1	–	***	84.2	80.6	87.9	0.267	0.214	0.193	0.189	0.237	0.190	0.020	0.038
DBP4_DBP5_DBP11	–	***	78.9	77.4	83.2	0.427	0.230	0.375	0.291	0.477	0.232	0.149	0.148
DBP3_DBP4_DBP12	***	–	94.7	62.4	81.0	0.336	0.232	0.328	0.241	0.363	0.207	0.102	0.136

Mean values and SD of each group; NE, not expressed, ***p < 0.00003 (BF).

Most significant results (*p* < BF) applying non-rotated adapted SPPA3 analysis were achieved by SBP5_SBP5_SBP2 for the group test CH and PIH vs. PE (test VIII) and SBP5_SBP5_SBP2 for test IX obtaining an AUC of 86.6%, sensitivity of 80.6%, and a specificity of 78.9%, respectively. The rotated version of adapted SPPA3 achieved most significant results by SBP2_SBP3_SBP1 for test IX (87.9%/80.6%/84.2%) and DBP3_DBP4_DBP12 for the test VIII (81%/94.7%/62.4%).

Multivariate discriminant analysis was applied to non-rotated and rotated predefined as well as adapted SPPA sets of two indices including one multivariate and one univariate SPPA3 index, respectively. Here, the group test between CH and PIH vs. PE (Test VIII) yielded best results for SBP5_SBP5_SBP2 and BBI3_DBP5_RESP2 (91.2%/84.2%/81.5%) applying non-rotated adapted SPPA3 as well as for SBP5_SBP5_SBP11 and BBI9_SBP6_DBP12 (91%/78.9%/88.9%) applying rotated adapted SPPA3. The group test between PREG, CH, and PIH vs. PE (Test IX) revealed the best results for SBP2_SBP3_SBP1 and SBP6_DBP7_RESP10 (91.7%/100%/81.7%) applying rotated adapted SPPA3 as well as for SBP5_SBP5_SBP2 and SBP8_DBP9_RESP9 (90.3%/89.5%/83.9%) applying non-rotated adapted.

The numbers of highly significant indices regarding the coarsely segmented cubic box model (6 × 6 × 6) are shown within Table 7 (rotated versions) and Table 8 shows the non-rotated versions. The tables include the number of significant cubelets within the whole cubic box model for each group test. The marked results show the couplings yielding most significant cubelets for each group test. The non-rotated predefined SPPA3 revealed most significant cubelets especially for couplings between the three different systems (BBI, SBP, and RESP) except test II, IV, V, and VI. The highest number of significant cubelets was calculated from test IV (PREG vs. PE) and IX (PREG, CH and PIH vs. PE).

**Table 7:** **Coarsely segmented cubic box model applying the rotated SPPA3 methods**.

Test	I	II	III	IV	V	VI	VII	VIII	IX	X
**Adapted SPPA3**
BBI_DBP_RESP	7	4	3	12	0	0	4	2	8	2
BBI_SBP_DBP	3	13	3	4	0	1	7	5	6	7
BBI_SBP_RESP	5	3	10	7	0	0	5	1	5	6
SBP_DBP_RESP	4	1	2	7	0	0	5	6	7	1
**Predefined SPPA3**
BBI_DBP_RESP	4	0	1	13	0	1	3	7	14	5
BBI_SBP_DBP	26	0	8	18	1	3	5	8	19	7
BBI_SBP_RESP	5	1	3	17	0	1	7	7	16	11
SBP_DBP_RESP	5	8	8	23	1	4	8	5	21	19

*Number of significant cubelets (*p* < 0.01) for the two investigated approaches of SPPA3 applying all kinds of couplings; the coupling yielding the most significant cubelets is marked*.

**Table 8:** **Coarsely segmented cubic box model applying the non-rotated SPPA3 methods**.

Test	I	II	III	IV	V	VI	VII	VIII	IX	X
**Adapted SPPA3**
BBI_DBP_RESP	11	2	2	7	1	2	4	3	6	0
BBI_SBP_DBP	18	0	4	12	0	7	10	13	11	1
BBI_SBP_RESP	17	3	1	13	0	6	10	12	15	0
SBP_DBP_RESP	13	1	1	12	0	8	10	13	13	0
**Predefined SPPA3**
BBI_DBP_RESP	12	0	9	44	3	8	10	21	42	15
BBI_SBP_DBP	14	7	11	38	2	9	8	17	36	22
BBI_SBP_RESP	16	5	20	43	0	5	17	21	44	38
SBP_DBP_RESP	10	9	9	30	5	12	12	12	26	27

*Number of significant cubelets (*p* < 0.01) for the two investigated approaches of SPPA3 applying all kinds of couplings; the coupling yielding the most significant cubelets is marked*.

The highly segmented cubic box model (Table 9) showed most discrimination power for non-rotated predefined SPPA3 applying couplings between two systems (Test VIII – BBI6_SBP6_DBP4; 84.6%/84.2%/81.5%) and the non-rotated adapted SPPA3 applying couplings between two different systems (Test IX – BBI2_SBP3_DBP2; 81.4%/84.2%/81.5%).

**Table 9:** **Best result of each approach including rotated and non-rotated (ROT – rotation) methods for the coarsely segmented cubic box model (6 × 6 × 6 cubelets)**.

Meth	ROT	Test	Index	*U*-test	SENS	SPEC	AUC	VIII: CH, PIH; IX: PREG, CH, PIH		PE	
								Mean	SD	Mean	SD
Predefined SPPA3	Yes	VIII	BBI3_DBP4_RESP3	**	52.6	96.3	76.1	0.980	4.717	10.384	16.134
			BBI1_SBP4_DBP2	**	31.6	96.3	64.1	0.008	0.043	1.570	4.053
			BBI3_SBP4_RESP3	***	73.7	88.9	81.7	1.706	8.597	9.447	15.838
			SBP1_DBP1_RESP2	**	36.8	100.0	68.4	0.000	0.000	1.412	3.214
		IX	BBI3_DBP4_RESP3	***	57.9	91.4	76.6	0.608	3.031	10.384	16.134
			BBI1_SBP4_DBP2	***	31.6	98.9	65.3	0.002	0.023	1.570	4.053
			BBI3_SBP4_RESP3	***	73.7	91.4	83.5	0.614	4.722	9.447	15.838
			SBP1_DBP1_RESP1	***	31.6	100.0	65.8	0.000	0.000	3.094	6.471
	No	VIII	BBI6_DBP6_RESP3	***	100.0	59.3	80.9	2.503	4.649	0.012	0.040
			BBI6_SBP6_DBP4	***	84.2	81.5	84.6	5.817	7.998	1.523	6.244
			BBI3_SBP4_RESP4	***	68.4	92.6	80.2	1.335	6.573	7.906	13.315
			SBP1_DBP1_RESP1	***	68.4	92.6	80.1	0.229	0.983	13.698	22.658
		IX	BBI2_DBP5_RESP3	***	63.2	92.5	77.2	0.156	1.305	5.555	11.816
			BBI1_SBP4_DBP1	***	63.2	92.5	78.7	0.290	2.569	4.771	8.306
			BBI2_SBP5_RESP4	***	57.9	95.7	76.9	0.009	0.049	0.535	1.194
			SBP3_DBP1_RESP6	***	63.2	96.8	79.9	0.092	0.760	2.790	5.497
Adapted SPPA3	Yes	VIII	BBI5_DBP3_RESP6	**	84.2	70.4	78.0	7.329	1.967	10.051	2.923
			BBI4_SBP4_DBP2	**	100.0	59.3	77.5	0.102	0.145	0.006	0.018
			BBI5_SBP3_RESP6	**	73.7	70.4	74.5	5.195	3.473	7.783	2.585
			SBP5_DBP2_RESP3	**	84.2	77.8	79.1	0.190	0.216	0.041	0.081
		IX	BBI1_DBP6_RESP1	***	15.8	100.0	57.9	0.000	0.000	0.009	0.022
			BBI5_SBP4_DBP6	**	73.7	72.0	76.0	0.587	0.470	1.339	1.517
			BBI6_SBP1_RESP5	***	15.8	100.0	57.9	0.000	0.000	0.009	0.021
			SBP5_DBP2_RESP3	***	84.2	73.1	77.7	0.221	0.263	0.041	0.081
	No	VIII	BBI3_DBP3_RESP6	**	78.9	77.8	80.7	0.427	0.232	0.182	0.190
			BBI2_SBP3_DBP2	**	84.2	81.5	81.4	0.562	0.306	0.216	0.230
			BBI2_SBP2_RESP1	***	89.5	66.7	80.4	0.168	0.210	0.015	0.032
			SBP4_DBP2_RESP1	***	63.2	92.6	82.7	1.128	0.434	0.569	0.416
		IX	BBI3_DBP3_RESP6	**	73.7	68.8	74.9	0.378	0.241	0.182	0.190
			BBI2_SBP4_DBP2	***	94.7	54.8	75.7	0.069	0.090	0.003	0.013
			BBI6_SBP1_RESP1	***	15.8	100.0	57.9	0.000	0.000	0.009	0.021
			SBP2_DBP4_RESP6	***	15.8	100.0	57.9	0.000	0.000	0.025	0.084

Mann–Whitney *U*-test as well as discriminant analysis (SENS – sensitivity, SPEC – specificity, AUC) of the most important group tests (CH and PIH vs. PE – Test VIII and PREG, CH and PIH vs. PE – Test IX) including mean and SD of applied groups; **p < 0.01, ***p < 0.00023 (BF); highest AUC values (AUC > 80%) are marked.

## 4 Discussion

In this study, we introduced the new SPPA3 method, which is a dimensional enhancement of the 1D SPPA introduced by Voss et al. (2010a). Hereby, SPPA3 investigates the three-dimensional phase space retaining non-linear features of coupled systems’ dynamics.

SPPA3 implies a range of different approaches, whereby the position of the cloud of points was chosen as rotated or non-rotated version. Further on, a highly segmented cubic box model with equal dimensioned 12 × 12 × 12 cubelets was developed where the axis sizes of the single cubelets could be defined in two different ways: the first version is where the axis sizes are predefined according to physiological dimensions (Table 2). The second version is where the axis sizes are determined as SD of the related time series. For each cubelet, the relative percentage of occurrence of points is calculated getting the single percentages (probabilities of occurrences). Furthermore, a coarsely segmented cubic box model was performed summing up single percentages of four neighboring cubelets of each axis leading to a coarsely segmented 6 × 6 × 6 cubic box model (reduction by a factor of 2^3^).

It could be demonstrated that SPPA3 is suitable for analyzing either the time correlation within one system (univariate) or the instantaneous coupling between two or three different (sub)systems (multivariate). In nearly all tests, highly significant differences between the patient groups were found.

Voss et al. (2010b) showed that the alterations of the interaction between BBI and SBP differ significantly between hypertensive groups applying JSD, which is demonstrably capable to differentiate the autonomic regulation between hypertensive pregnancy disorders and PE. In this study, we could confirm this cardiovascular alteration, and furthermore, considered and established the influences of normal and hypertensive pregnancy disorders on the cardiorespiratory system.

Comparing the number of highly significant cubelets (Tables 3 and 4), the combination of non-rotated position of the cloud of points with predefined dimensions of each cubelet in the cubic box model seemed to be the optimum SPPA3 method differentiating between non-pregnant and pregnant women, pregnant women, and hypertensive pregnancy disorders as well as hypertensive pregnancy disorders and PE.

For clinicians, the differentiation between preeclamptic and hypertensive as well as between preeclamptic and all other pregnant women is the most important tasks (tests VIII and IX in Tables 5 and 6). Therefore, only those tests were further considered.

The most discrimination power differentiating between preeclamptic and all other pregnant women could be reached with two (BBI, SBP, DBP; AUC = 82.9%) and between preeclamptic and other hypertensive pregnant women with three (BBI, SBP, RESP; AUC = 82.8%) coupled systems applying non-rotated adapted SPPA3. This is in accordance with the recently introduced SPPA and bivariate SPPA (BSPPA) methods (Voss et al., 2010a, 2014). In Voss et al. (2014), the best discrimination power differentiating hypertensive pregnancy disorders and PE was achieved in the right part of row 9 (AUC = 75.8%) quantifying couplings between BBI and SBP time series. When evaluating these couplings, it was advisable to observe specific regions of the cloud of points as well as total row and column percentages. For this type of observation, two segmentation algorithms were introduced: (a) the summation of all single percentages (1–12) of one row or column; and (b) the summation of half of them (e.g., row9_right) for a more detailed segmentation. In this study, we could improve the accuracy to an AUC of 82.8% by expanding the dimension and including a third coupling system – the cardiorespiratory regulation (BBI5_SBP7_RESP12). This leads to significant increases of mean BBI and SBP in combination with very high respiration rates (very short breathing cycles) in preeclamptic women. This is in accordance with the study of Riedl et al. (2010) who could show that this cardiorespiratory regulation is impaired in PE and that the non-linear form of the respiratory influence on the heart rate is significantly different between PE and PREG.

Furthermore, we could improve the accuracy of BSPPA differentiating between PE and other hypertensive pregnancy disorders from 75.8 to 82.9% AUC with DBP regulation as third dimension (BBI9_SBP6_DBP11).

Additionally, the coupling of SBP with all other included physiological systems (BBI, SBP, and RESP) revealed higher significances than that with DBP (BBI, DBP, and RESP) in all investigated methods of SPPA3 except rotated predefined SPPA3. The cubelets representing coupling combinations including SBP are situated in the outer regions of the cubic box model of rotated and non-rotated adapted SPPA3.

Interestingly, univariate SPPA3 yield to more discrimination power than multivariate SPPA3 especially for non-rotated and rotated adapted SPPA3. Therefore, the SBP is of particular importance reaching AUC values of 86.6% with the non-rotated and 87.9% with the rotated version of adapted SPPA3. The reason for this could be that the time correlations regarding univariate SPPA3 provide more detailed information about the investigated system. Multivariate SPPA3 only represents the coupling of the systems in a specific time instant while the univariate SPPA3 considers the inner system’s coupling over a lag of three successive beats. Therefore, it would be of interest to enhance the multivariate SPPA3 methods with a time correlation approach.

Seeck et al. (2011) applied SPPA to differentiate between women with hypertensive pregnancy disorders and PE investigating BBI as well as NIBP signals. The optimum set of indices consisting of two SPPA indices (SBP) led to an AUC of 83.6%. In our study, this was increased to 91.2% applying one time correlating (univariate) SPPA3 index of SBP time series and one non-rotated adapted SPPA3 index including the three different coupling systems (BBI, DBP, RESP). This result could be improved discriminating between all pregnant women (PREG + CH + PIH) and PE reaching an AUC of 91.7% (SBP, DBP and RESP).

Obviously, the coarsely segmented cubic box model (6 × 6 × 6 cubelets) showed more highly significant parameter compared to highly segmented cubic box model of SPPA3 (12 × 12 × 12 cubelets). Here again, we considered only the clinically relevant tests VIII and IX (Table 9). Differentiating between PE and the other hypertensive disorders as well as between PE and all other pregnancies revealed an AUC of 83.5% (BBI, SBP, RESP), respectively, an AUC of 84.6% (BBI, SBP, DBP) applying the predefined SPPA3 method. The reason for this slight increase of accuracy could be that boundary value problems are decreased by the reduction of the number of cubelets. This coarsely segmented cubic box model seems to be a more robust method. Therefore, in further studies, it is suggested to analyze also the univariate SPPA3 in combination with multivariate classification on the basis of this coarsely segmented cubic box model.

Summarizing our findings, SBP seems to be the primary, superior factor influencing the results of all introduced methods of SPPA3. The other investigated coupled time series (BBI, DBP, and RESP) provide additional discriminant power in all group tests applying both SPPA3 methods (adapted or predefined).

All expressed results are based on the most significantly discriminating index of each investigated SPPA3 method. Further studies should prove if the other highly significant indices can further improve the presented results.

One limitation of this study is the low sampling frequency (200 Hz) of blood pressure time series and another one the extracting of heart rate from the blood pressure curves that could lead to lower precision in estimating BBI using the “BeatFast” pattern recognition software package. In further studies, the BBIs should be extracted directly from high resolution ECG.

In conclusion, non-rotated adapted SPPA3 demonstrates a new and useful approach to analyze couplings between two and three different time series and univariate time courses. SPPA3 offers a new tool for enhanced risk stratification in pregnant women suffering from PE.

## Conflict of Interest Statement

The authors declare that the research was conducted in the absence of any commercial or financial relationships that could be construed as a potential conflict of interest.
